# The Use of Targeted Next Generation Sequencing to Explore Candidate Regulators of TGF-β1’s Impact on Kidney Cells

**DOI:** 10.3389/fphys.2018.01755

**Published:** 2018-12-10

**Authors:** Bo Wang, Guanyu Ji, Haroon Naeem, Junwen Wang, Phillip Kantharidis, David Powell, Sharon D. Ricardo

**Affiliations:** ^1^Department of Anatomy and Developmental Biology, Monash Biomedicine Discovery Institute, Monash University, Clayton, VIC, Australia; ^2^Shenzhen E-GENE Tech Co., Ltd., Shenzhen, China; ^3^Monash Bioinformatics Platform, Monash University, Clayton, VIC, Australia; ^4^Monash Biomedicine Discovery Institute, Monash University, Clayton, VIC, Australia; ^5^Department of Diabetes, Monash University, Clayton, VIC, Australia

**Keywords:** transforming growth factor-beta, diabetes, fibrosis, RNA-Seq, DNA methylation

## Abstract

**Aims/Hypothesis:** Transforming growth factor-beta (TGF-β1) plays an important regulatory role in the progression of chronic kidney failure. Further, damage to kidney glomerular mesangial cells is central to the progression of diabetic nephropathy. The aim of this study was to explore the genetic associations between mRNA, microRNA, and epigenetics in mesangial cells in response to TGF-β1.

**Methods:** The regulatory effects of TGF-β1 on mesangial cells were investigated at different molecular levels by treating mesangial cells with TGF-β1 for 3 days followed by genome-wide miRNA, RNA, DNA methylation, and H3K27me3 expression profiling using next generation sequencing (NGS).

**Results:** Our results provide the first comprehensive, computationally integrated report of RNA-Seq, miRNA-Seq, and epigenomic analyses across all genetic variations, confirming the occurrence of DNA methylation and H3K27me3 in response to TGF-β1. Our findings show that the expression of KLF7 and Gja4 are involved in TGF-β1 regulated DNA methylation. Our data also provide evidence of the association between epigenetic changes and the expression of genes closely related to TGF-β1 regulation.

**Conclusion:** This study has advanced our current knowledge of mechanisms that contribute to the expression of TGF-β1-regulated genes involved in the pathogenesis of kidney disease. The molecular underpinnings of TGF-β1 stimulation of kidney cells was determined, thereby providing a robust platform for further target exploration.

## Introduction

The transforming growth factor-beta (TGF-β) superfamily of regulatory proteins consists of secreted peptides that are important to mammalian development and homeostasis. However, TGF-β1 plays a critical role in the pathogenesis of both acute and chronic kidney disease (CKD), since long terms TGF-β1 overproduction can lead to end-stage diabetic nephropathy. TGF-β1 also plays a central role in the development of kidney fibrosis and inflammation through its downstream signaling cascades, which activate cellular pathologic mechanisms underlying the progression of renal inflammation and fibrosis (Border et al., 1990; Basile, 1999; Oxburgh et al., 2004). The precise mechanisms that mediate TGF-β1-induced fibrosis, however, are not well understood.

Awareness of the critical role of epigenetic and small RNA-related mechanisms in the regulation of gene expression in both tissue homeostasis and disease is increasing. Gene expression is regulated post-transcriptionally by miRNAs, which are short, non-coding RNAs that bind to the 3′-UTR (untranslated region) of target mRNAs and repress protein production by destabilizing the mRNA, thus silencing translation (Mehta and Baltimore, 2016). DNA methylation, on the other hand, is a common epigenetic mechanism used by cells to repress gene expression. DNA methylation is also critical to the regulation of RNA expression and is an important component of numerous cellular processes that are essential for kidney development and tissue inflammation in acute and chronic diseases (Bomsztyk and Denisenko, 2013; Woo et al., 2014; Marumo et al., 2015). Histone modification, which involves the transfer of methyl groups to the histone proteins that comprise the nucleosome, is a separate epigenetic modification that plays a role in kidney development and disease (Chen and El-Dahr, 2013; Wang et al., 2014). To this end, determining the specific functions of histone subtypes in the development of kidney disease is important for identifying new therapeutic targets.

Histone H3K27 is involved in the regulation of gene transcription. Specifically, trimethylated H3K27 (H3K27me3) is strongly associated with inactive gene promoters and the inhibition of transcription regulated by enhancer of zeste homolog 2 (EZH2), a specific histone methyltransferase for H3K27me3 (Simon and Lange, 2008). Due to its marked and predictable effect on gene expression, H3K27me3 is also an important epigenetic marker. However, recent studies have indicated that H3K27me3 plays an opposing role in the development of tissue fibrosis (Siddiqi et al., 2016; Zhou et al., 2016), prompting further investigation of the function of this novel regulator in the regulation of kidney fibrosis.

The advent of next generation sequencing (NGS) technologies has enabled genetic nephrology research to advance beyond the analysis of single genes to the simultaneous investigation of hundreds of genes and entire pathways. NGS technologies are suitable for mapping the transcriptome (RNA-Seq), small RNAs (miRNA-Seq), histone modifications using chromatin immunoprecipitation (ChIP) assays (ChIP-Seq), and DNA methylation using methylated DNA immunoprecipitation technology (MeDIP-Seq). Additionally, RNA-Seq and miRNA-Seq are used to analyze mRNA and miRNA expression levels as well as splicing variants and non-coding RNAs on a genome-wide scale, whereas ChIP-Seq and MeDIP are used to identify genome-wide transcriptional DNA-binding sites, which are known to regulate gene expression in both intragenic and intergenic regions. The combination of the miRNA-Seq, ChIP-Seq, MeDIP-Seq, and RNA-Seq approaches facilitates the identification of novel transcriptional mechanisms that may play important roles in various diseases. Recently, studies involving NGS technologies have investigated the effects of TGF-β on different cell types (Brennan et al., 2012; Tian et al., 2015). However, these studies were independently assessed and did not involve coordinated investigations to evaluate the genome-wide effects of TGF-β1 expression, especially in kidney cells.

Accordingly, we investigated the immediate transcriptional responses induced by TGF-β1 and specifically determined changes in mRNA, miRNA, histone (H3K27me3), and DNA methylation in primary mesangial cells. We report TGF-β1-induced changes in the expression of genes involved in metabolic and endocrine diseases and fibrosis that were associated with genome-wide changes in histone and DNA methylation. NGS was used to demonstrate the remarkable complexity of the multiple omics in kidney mesangial cells, which has not been previously demonstrated. A comparative analysis of dataset from gene expression, methylation of chromatinized DNA, and miRNA expression was elucidated when the regulatory mechanisms were considered central to TGF-β1 responses.

## Materials and Methods

### Flowchart of Analytic Methods

**FIGURE 1:**
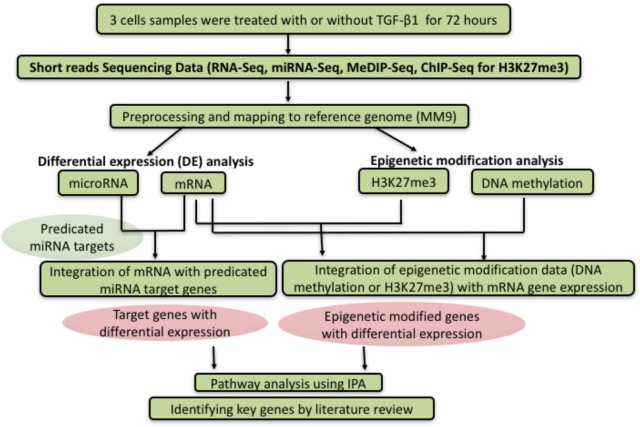
Flowchart of analytic methods. After sequencing the expression level of miRNA, RNA, DNA methylation, and H3K27me3 between control and TGF-β1 treated samples, the datasets were analyzed to investigate the correlation between different genetic or epigenetic level, following by analysis and interpretation through the pathway analysis or literature review.

Figure 1 summarized the flowchart of analytic methods for this study. More details are described in “Materials and Methods” and “Results”.

### Cell Culture and TGF-β1 Treatment

Mouse primary mesangial cells (provided by Professor Joseph Forbes, University of Queensland, Australia) (Hagiwara et al., 2018) were cultured in Dulbecco’s Modified Eagle Medium with the addition of high glucose (25.5 mM) containing 20% fetal bovine serum (GIBCO). The cells used in these experiments were used between passages 3 and 6 and were treated with 10 ng/ml TGF-β1 for 72 h.

### miRNA-Seq and RNA-Seq

Total RNA was isolated from the mesangial cells using TRIzol^®^ (Invitrogen) per the manufacturer’s instructions. The samples were collected from each group (TGF-β1 and control cells) in triplicate and RNA sequencing (RNA-Seq) and miRNA sequencing (miRNA-Seq) were performed using the Illumina HiSeq^TM^ 2000 sequencing system [Beijing Genomics Institute (BGI) Inc., Shenzhen, China]. An analysis of differential miRNA (DE-miRNA) and RNA (DE-genes) expression between samples from TGF-β1-treated and control cells was then conducted.

Low-quality reads from the RNA-Seq results were removed from the data set and the clean reads were mapped to the mouse reference genome (MM9) using TopHat (version 2.0.6) (Trapnell et al., 2009) with the default settings. Cufflinks (version 2.0.2) was used to estimate the fragments per kilobase of exon per million fragments mapped (FPKM) values (Trapnell et al., 2010), and genes with an FPKM value ≥3 were considered expressed, as previously described (Mortazavi et al., 2008). Next, the differential expression of genes between the TGF-β1-treated and control samples was reported as fold change (FC) with associated p-values calculated based on a negative binomial distribution, and evaluated using the DESeq R Bioconductor package (Anders and Huber, 2010). The approach was well suited for experiments involving count data (read counts), and the method estimated variance in a local fashion for varying signal strength (Trapnell et al., 2010).

The annotation of the miRNA reads from miRNA-Seq results was performed as previously described (Tan et al., 2014). Briefly, clean reads were obtained after trimming the 5′ and 3′ adaptors and eliminating contaminants and inadequate (<18 nt) and low-quality reads. The trimmed sequences were compared against known miRNAs released from miRBase version 17 (Griffiths-Jones et al., 2006) using the Bowtie algorithm (Langmead et al., 2009). The expression of each miRNA was calculated as transcripts per million reads (TPM), and log_2_ fold changes (log_2_FC) between the TGF-β1-treated and control groups were calculated based on normalized read counts.

Significant differences in the expression of miRNAs between the TGF-β1-treated and control samples were determined using the DESeq (Anders and Huber, 2010) package in R. The threshold for differentially expressed miRNAs was set at an FDR *p*-value <0.05 and | log_2_ FC| >2. Additionally, we analyzed the expression pattern of DE-genes by comparing the endogenous expression levels of genes between the groups. The quintile analysis ranked the gene expression levels from low expression (bottom 20% in Rank 1) to highly expressed (top 20% in Rank 5).

### Analysis of the miRNA–mRNA Relationship

Putative target genes of the differentially expressed mouse miRNAs were predicted both using TargetScan (Lewis et al., 2005) and miRDB (Wong and Wang, 2015) for overlapping targets by miRWalk (Dweep and Gretz, 2015). Besides traditional 3′UTR located miRNA targets, the CDS- and 5′UTR-located miRNA targets are predicted (Hausser et al., 2013; Gu et al., 2014). For each miRNA–target pair, we calculated the FC1/FC2 value, which represented the strength of the DE-miRNA-DE-gene relationship, where FC1 indicated the log_2_FC in the DE-gene (calculated between the TGF-β1-treated and control samples), and FC2 indicated the log_2_FC of the DE-miRNA (Zhuang et al., 2015).

### MeDIP-Seq for DNA Methylation Analysis

The raw reads (three replicates per group) were obtained by Illumina sequencing. Then we get the clean reads from the data set by filtering reads contained the adaptor sequence, or the reads which had low quality and N bases occupy more than 10% of the reads length. The clean reads were aligned to the mouse reference genome (MM9) using the Burrows-Wheeler Aligner (BWA) (Li and Durbin, 2010), allowing up to two nucleotide mismatches to the reference genome per seed and returning only uniquely mapped reads. Replicate sequencing reads (i.e., reads with the same starting position) were counted only once.

Uniquely mapped reads were used to analyze the peaks based on a defined model of ChIP-Seq data (MACS2) (Liu, 2014). Peaks with a false discovery rate (FDR) less than 0.01 were used for further analysis, and all the other parameters were used as default. Based on our results, we found 81,473, 90,337, and 100,449 peaks in the control samples and 101,629, 100,379, and 105,520 peaks in the TGF-β1-treated samples. After merging the replicate samples, however, we found 200,097 peaks in the control and 207,163 peaks in TGF-β1-treated samples. Most of the peaks in the biological replicates overlapped with the merged peaks. Thus, we determined that the data may not be saturated and selected data for the next differential analysis. The peaks from the TGF-β1-treated and control samples were then merged as candidate differentially methylated regions (CDMRs). The reads were counted for each of the CDMRs, and the DESeq R Bioconductor package (Anders and Huber, 2010) was used to identify CDMRs with a FDR < 0.01 and | log_2_ FC| >2 as final differentially methylated regions (DMRs).

Read distributions were normalized using the Reads Per Kilobase per Million (RPKM) mapped reads strategy in 100 bp bins, followed by the analysis of the immunoprecipitation-based DNA methylome using a method based on a Bayesian deconvolution strategy (Down et al., 2008). A prior report (Chavez et al., 2010) demonstrated that this normalization strategy improves the correlation to bisulfite sequencing data. The paired reads were not extended and were placed in bins of 100 bp across the genome. The values of each bin were used to determine the read density or read distribution on the gene element regions.

### Analysis of H3K27me3 Methylation by ChIP-Seq

Triplicate ChIP-Seq analyses were performed in each group (TGF-β1-treated and control samples) using an antibody that recognized the trimethylation of lysine 27 on histone H3 (H3K27me3). Data were processed and analyzed as described for the MeDIP data with the exception that the broad peak mode was used for peak calling. Briefly, three samples were merged from each group and peak calling was performed using MACS. The differentially methylated peaks were then identified, followed by MeDIP analysis pipelines. The distribution of reads for each H3K27me3 sample was normalized as read density values using the RPKM strategy.

### Ingenuity Pathway Analysis

Pathway analysis was performed using the Ingenuity Pathway Analysis (IPA) tool to elucidate the underlying biological function of the differentially expressed genes identified.

### Raw Data Access

All sequencing data had been deposited at NCBI Gene Expression Omnibus (GEO), and the GEO number is GSE121702.

## Results

### Gene Expression Patterns in Mesangial Cells Following TGF-β1 Stimulation

To identify the signaling pathways activated in mesangial cells following treatment with TGF-β1, changes in global gene expression were examined by NGS. We identified 5,140 genes with significantly different expression between the TGF-β1-treated and control samples (2,600 upregulated and 2,540 downregulated at a FDR of 5%). The top 10 upregulated and downregulated genes ranked by FC are shown in Table 1 (see Supplementary Table S1 for full list of DE-genes). Among these genes, *Tnfrsf11b*, *Rxfp3*, *MMP9*, *Syn1*, *IL6*, and *Megf6* are known to regulate fibrosis and inflammatory pathways. The functions of other genes listed in Table 1 remain unclear for their role in fibrosis and inflammation regulatory pathways.

**Table 1:** The top-10 DE-genes with dys-regulated expression.

Gene ID	Symbol	Log_2_ fold change
**Top 10 up-regulated genes**
NM_008764	Tnfrsf11b	17.29212337
NM_178717	Rxfp3	15.24104987
NM_013599	Mmp9	15.20316701
NM_013680	Syn1	15.10553835
NM 031168	ll6	15.01685333
NM_001162977	Megf6	15.00097206
NM_007811	Cyp26a1	14.75727491
NM_009612	Acvrl1	14.55548557
NM_153505	Nckap1l	14.54824345
NM_010188	Fcgr3	14.43942319
**Top 10 down-regulated genes**
NM-029550	Keg1	-17.01421953
NM_144834	Serpina1O	-16.53461514
NM_010074	Dpp4	-16.0033186
NM_007831	Dcc	-15.83524901
NM_001037915	Ripply1	-15.65286525
NM_028880	Lrrtm1	-15.54897139
NM_001038699	Fn3k, transcript variant 2	-15.47795143
NM_145835	Lctl	-15.37077912
NM_022014	Fn3k, transcript variant 1	-15.20434621
NM_146240	Rassf9	-15.19399183

*The list of genes with most folder changes, including up-regulated and down-regulated genes.*

To identify signaling pathways involved in kidney cell damage following TGF-β1 treatment, we conducted an IPA of the full DE-gene list, which clearly revealed the activation of the integrin, epithelial adherens junction, Wnt/β-catenin, remodeling of epithelial adherens junctions, and Rho Family GTPase signaling pathways. Other known fibrosis and inflammation-related pathways were likewise identified including RhoGDI, PI3K/AKT, TGF-β, ERK/MAPK, PTEN, mTOR, RhoA, and VEGF (Table 2). The full IPA list is provided in Supplementary Table S2.

**Table 2:** Ingenuity Pathway Analysis (IPA) for differentially expressed genes.

Rank	Ingenuity canonical pathways	-log(B-H *p*-value)	Inflammation/fibrosis related
	**Top 10 pathways**		
1	Axonal guidance signaling	8.58	
2	Superpathway of cholesterol biosynthesis	6.6	
3	Integrin signaling	6.6	Both
4	Germ cell-sertoli cell junction signaling	6.6	
5	Epithelial adherens junction signaling	6.6	Fibrosis
6	Cholesterol biosynthesis I	5.9	
7	Cholesterol biosynthesis II (via 24,25-dihydrolanosterol)	5.9	
8	Cholesterol biosynthesis III (via desmosterol)	5.9	
9	Wnt/β-catenin signaling	5.28	Both
10	Remodeling of epithelial adherens junctions	5.14	Fibrosis
	**Other important fibrosis/inflammation related pathway**		
18	Signaling by rho family GTPases	4.09	Both
33	PI3K/AKT signaling	2.53	Both
36	TGF-β signaling	2.52	Both
48	ERK/MAPK signaling	2.18	Both
49	PTEN signaling	2.18	Both
53	mTOR signaling	2.06	Both
74	VEGF signaling	1.58	Both

*IPA shows the top 10 dys-regulated pathways and other inflammation/fibrosis-related pathways with high ranking. “-log(B-H p-value) is -1 ^∗^ log(p-value) after Benjamini-Hochberg multiple hypothesis correction.”*

### Comparison of Gene Abundance Using Quintile Analysis

A separate method for identifying potential TGF-β targets among DE-genes based on the ranking of endogenous gene abundance by quintile analysis (Al Seesi et al., 2014) was also applied. The genes were sorted by the average gene expression level based on the associated FPKM values. The endogenous expression of DE-genes was then characterized, with the relationship between TGF-β1 treatment and controls determined by dividing DE-genes into expression level quintiles in which quintile 1 contained the genes with the lowest expression levels (Rank 1) and quintile 5 contained the genes with the highest expression levels (Rank 5). The ranking of all DE-genes (Rank 1–5) is shown in Supplementary Table S3.

**FIGURE 2:**
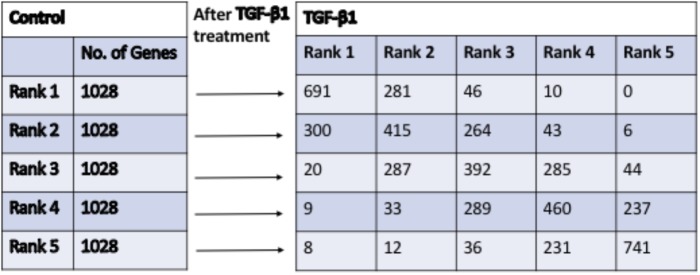
The quintile distribution of DE-genes changes after treatment with TGF-β1, in comparison to control. The genes expression levels were divided equally into 5 ranks from 1 to 5 (from low to high) in Control and TGF-β1 groups. The genes from ranks (1–5) in Control group moved into different ranks (1–5) in TGF-β1 group.

The results of the quintile analysis in Figure 2 showed the distribution of genes with the lowest expression (bottom 20%, 1,028 genes in Rank 1) to genes with highly expressed (top 20%, 1,028 genes in Rank 5) in the control and the number of genes that moved from one rank in control to other in TGF-β1-treated samples (Rank 1 to Rank 5). The DE-genes with the most significant rank changes, including genes found in Rank 5 in the controls that appeared in Rank 1 in the TGF-β-treated samples, are listed in Table 3. Interestingly, none of the DE-genes that appeared in Rank 1 in the controls were found in Rank 5 in the TGF-β-treated samples. We listed all 10 genes from Rank 1 in the controls to Rank 4 in the TGF-β-treated samples and 5 genes from Rank 1 in the controls to Rank 4 in the TGF-β-treated samples. For example, among the genes on the list, the matrix Gla protein (Mgp) expression is reduced by TGF-β1 treatment (Zhao and Warburton, 1997). Conversely, cadherin 10 (Cdh10) acts as a biomarker of TGF-β-induced epithelial-mesenchymal transition (Johansson et al., 2015), in which TGF-β1 play important role (Xu et al., 2009). The collagens are well known to be up-regulated by TGF-β. When the genes in Table 3 were compared to the genes in Table 1, there were no overlapping genes. Interestingly, Rassf9 (RAS association domain family member 9 in Table 1) and Rasl11a (a small GTPase protein with a high similarity to Ras in Table 3) were both down-regulated significantly by TGF-β1, suggesting the involvement of Ras pathway effectors in TGF-β1 regulation. The results of the quintile analysis suggested an alternative to traditional FC analysis for exploring significant candidate genes.

**Table 3:** Comparison of DE-genes ranking based on endogenous expression in control and TGF-β1 samples. In the up-regulated genes, all genes from Rank 2 in control to Rank 5 in TGF-β1 and from Rank 1 to Rank 4 are listed.

Gene ID	Symbol	Rank in control	Rank in TGF-β1
NM_001004174	*AA467197*	1	4
NM_053080	*Aldh1a3*	1	4
NM_009865	*Cdh10*	1	4
NM_030209	*Crispld2*	1	4
NM_177914	*Dgkk*	1	4
NM_008216	*Has2*	1	4
NM_053105	*Klhl1*	1	4
NM_027551	*Klhl30*	1	4
NM_001001985	*Nat8l*	1	4
NM_175093	*Trib3*	1	4
NM_153543	*Aldh1l2*	2	5
NM_007735	*Col4a4*	2	5
NM_016919	*Col5a3*	2	5
NM_001081437	*Fbln2*	2	5
NM_023279	*Tubb3*	2	5
NM_139298	*Wnt9a*	2	5
NM_007428	*Agt*	5	1
NM_009778	*C3*	5	1
NM_016675	*Cldn2*	5	1
NM_011339	*Cxcl15*	5	1
NM_008597	*Mgp*	5	1
NM_026864	*Rasl11a*	5	1
NM_053116	*Wnt16*	5	1

*There are no genes from Rank 1 in control to Rank 5 in TGF-β1. In the down-regulated genes, all genes with changes from Rank 5 in control to Rank 1 TGF-β1 are listed.*

### miRNA Regulatory Circuits in Response to TGF-β1

miRNAs are known to be involved in TGF-β-regulated cellular pathophysiology (Butz et al., 2012). To investigate the regulatory effects of miRNAs, the miRNA profiles were analyzed based on | log2FC| >1 and FDR < 0.05. The list of the upregulated and downregulated DE-miRNAs between the control and TGF-β-treated samples are detailed in Table 4. Established TGF-β1-regulated miRNAs were identified among these DE-miRNAs with most significant change, including miR-96, and miR-130, which are known to be regulated by TGF-β1 (Castro et al., 2014; Siu et al., 2015).

**Table 4:** The differentially regulated miRNA after TGF-β1 treatment.

miRNA	Fold change	| log2FC| >l
**Up-regulated miRNA**
mmu-miR-127-3p	2.06	1.04
mmu-miR-181a-2-3p	2.08	1.06
mmu-miR-379-5p	2.22	1.15
mmu-miR-183-3p	2.39	1.25
mmu-miR-485-5p	2.42	1.27
mmu-miR-96-5p	2.56	1.36
mmu-miR-433-3p	2.77	1.47
**Down-regulated miRNA**
mmu-miR-200a-3p	0.10	3.36
mmu-miR-3099-3p	0.16	2.66
mmu-miR-503-5p	0.20	2.33
mmu-miR-181c-3p	0.23	2.13
mmu-miR-351-5p	0.24	2.04
mmu-miR-351-3p	0.26	1.94
mmu-miR-101a-3p	0.27	1.91
mmu-miR-27a-3p	0.29	1.80
mmu-miR-26a-5p	0.36	1.48
mmu-miR-130a-3p	0.41	1.29
mmu-miR-130b-3p	0.48	1.07
mmu-miR-532-5p	0.49	1.02

### Interactive Analysis of miRNA Target Genes and miRNA

Because miRNAs can post-transcriptionally regulate target mRNAs by inducing mRNA repression and decay, only the target miRNA–mRNA pairs that exhibited opposite changes in expression were investigated. We identified 122 mRNA targets for 11 miRNAs using predicted analysis based on 3′UTR, 5′UTR and coding DNA sequence (CDS) (Supplementary Table S4).

Total 11 DE-miRNAs that significantly correlated with multiple target DE-genes are shown in Table 5 with the number of target genes. Among the down-regulated miRNAs, miR-130 (a&b) (35 target genes among 93 genes) and miR-27a (21 genes/93 genes) target most genes and are involved in the TGF-β1 pathway. The target genes are listed in Supplementary Table S4. It has been reported that miR-130a regulated the sensitivity of TGF-β1 stimulation through Smad4 and miR-130b is involved in TGF-β1 pathway by targeting TGF type I receptor (TGF-βR1) (Li et al., 2013; Koga et al., 2015). miR-27a is reported to down-regulate TGF-β1 pathway through TGF-βR1 (Nlandu Khodo et al., 2012). Among the up-regulated miRNAs, miR-96, miR-485 and miR-433 have target genes, in which miR-96 and miR-433 are related to TGF-β1 pathway (Li et al., 2013; Siu et al., 2015).

**Table 5:** The enriched DE-miRNAs and the number of target DE-genes.

miRNA	Number of predicted target genes
**Down-regulated miRNA**
mmu-miR-532	4
mmu-miR-503	1
mmu-miR-351	12
mmu-miR-27a	21
mmu-miR-26a	9
mmu-miR-200a	11
mmu-miR-130a	12
mmu-miR-130b	23
**Up-regulated miRNA**
mmu-miR-433	7
mmu-miR-96	13
mmu-miR-485	14

The strength of miRNA-target gene relationship is based on the fold change value of DE-gene (FC1)/DE-miRNA (FC2), which is used in a previous publication (Zhuang et al., 2015) (see section “Materials and Methods” for details). The top 10 target DE-genes with relationship to specific DE-miRNAs ranked according to relationship strength are shown in Table 6. Among these correlated miRNAs, miR-130 family (Castro et al., 2014) and miR-26 (Koga et al., 2015) are involved in TGF-β1 pathway. Among the correlated target DE-genes, Nox4 is known as a regulator in kidney injury process (Nlandu Khodo et al., 2012).

**Table 6:** The top-10 miRNA gene targets based on value of miRNA-gene target relationship.

miRNA family member	miRNA regulation by TGF-β	Gene symbol	Gene regulation	Relationship
mmu-miR-351-5p	Down	Tnfsf4	Up	-6.67570546
mmu-miR-485-5p	Up	Atoh8	Down	-3.93438238
mmu-miR-130b-3p	Down	Pmepal	Up	-3.76454559
mmu-miR-485-5p	Up	Clic5	Down	-3.69006107
mmu-miR-26a-5p	Down	Cd200	Up	-3.38661889
mmu-miR-130a-3p	Down	Pmepal	Up	-3.113305
mmu-miR-130b-3p	Down	Nox4	Up	-2.51621015
mmu-miR-351-5p	Down	Sema4f	Up	-2.31908499
mmu-miR-96-5p	Up	Pard3b	Down	-2.28866038
mmu-miR-130a-3p	Down	Nox4	Up	-2.08092303

*Value of relationship = log_2_FC gene/log_2_FC miRNA.*

### Analysis of DNA Methylation in Response to TGF-β1 Treatment

Epigenetic modifications are critical to the development and functioning of the kidney. However, little is known about the epigenome of renal disease. Current studies have shown that epigenetic modifications that alter gene expression play an important regulatory role in fibrogenesis during CKD (Higgins and Murphy, 2014). To investigate the regulatory effect of TGF-β1 treatment on the epigenetic regulation of mesangial cell genes, we profiled the DNA methylation levels of mesangial cell genes in the presence and absence of TGF-β.

**FIGURE 3:**
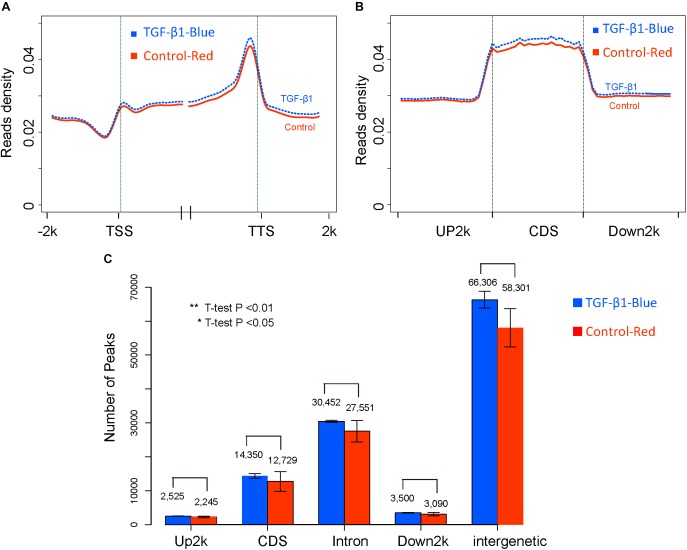
The global DNA methylation level between control and TGF-β1 samples. **(A)** The reads density in transcription start sites (TTS) and transcription termination sites (TTS). **(B)** The reads density across gene elements, including up2k, down2k and Coding DNA Sequencing (CDS). **(C)** The distribution of peaks through different gene elements.

Figure 3 shows that, globally, no significant difference between control and TGF-β1 treated samples was found at different gene elements level. Firstly, read density data was obtained by normalizing the read distribution using the RPKM mapped reads strategy in 100 bp bins (Down et al., 2008). The average read density across the entire genome was rounded up and down by 2k regions adjacent to the transcription start sites (TSS) and the transcription terminal sites (TTS) in the TGF-β-treated and control samples (Figure 3A). We also analyzed the average methylation levels across the gene elements (Figure 3B), including Up2k, Down2k, and the CDS. Both results revealed the trend of higher read densities in the TGF-β-treated group. However, the methylation features of peaks, which represent different gene elements (Figure 3C) by counting the gene peaks from each biological replicate, showed no significant differential expression across the genome. This suggests that TGF-β1 treatment does not cause significant global methylation in mesangial cells.

Next, we focused on modifications in the methylation of individual genes in response to TGF-β1 treatment, and identified 97 differentially methylated (31 hypermethylated and 66 hypomethylated) genes between the TGF-β1-treated and control groups (Supplementary Table S5). It is known that DNA methylation is an important epigenetic mechanism involved in the control of gene expression. Accordingly, we investigated how TGF-β1 targets specific genes by regulating DNA methylation by comparing DE-genes and methylated genes.

### Integrative Analysis of DNA Methylated Genes and DE-Genes

The integrated analysis of DNA methylation and DE-genes identified 32 genes (Table 7) with significant changes in their methylation status and gene expression measures (methylated DE-genes). Because DNA methylation can repress gene transcription, genes with altered DNA methylation and mRNA expression (negatively correlated) in the TGF-β1-treated compared to the control samples were retained for downstream analysis (Table 7). Seventeen genes exhibiting a negative correlation between gene expression and DNA methylation of DMR were identified. Among the 17 genes, 5 were downregulated and hypermethylated and 12 were upregulated and hypomethylated.

**Table 7:** Integrative analysis between DNA methylation and DE-genes.

Gene ID	Symbol	Gene expression fold change	Methylation fold change
**Positively correlated**		
NM_016687	*Sfrp4*	-4.59	-1.07348337
NM_026257	*Ubxn11*	-3.457	-1.03471761
NM_175199	*Hspa 12a*	-3.145	-1.46219747
NM_013460	*Adrald*	-2.851	-1.38134037
NM_008714	*Notch 1*	-1.568	-1.53733423
NM_009359	*Tex9*	-1.225	-1.12112261
NM_010806	*Afdn*	-0.776	-1.51548783
NM_146155	*Ahdd*	-0.726	-1.03777499
NM_029348	*Zbtb4*	-0.64	-2.07882427
NM_178878	*Hadha*	-0.555	-1.40843417
NM_173741	*Wdr24*	-0.537	-1.08835819
NM_133740	*Prmt3*	0.522	1.27886894
NM_001008421	*Nol10*	0.62	1.34590795
NM_008084	*Gapdh*	0.836	1.51507841
NM_007400	*Adam12*	3.106	1.18884493
**Negatively correlated**		
NM_008909	*Ppl*	-3.537	1.02814109
NM_009931	*Col4a1*	-1.111	1.18884493
NM_028200	*Vps9d1*	-0.89	1.32210035
*NM_033563*	*Klf7*	-0.633	1.48896494
NM_054044	*Adgra2*	-0.392	1.62011388
NM_009211	*Smarcd*	0.608	-1.04303283
NM_172585	*Larp4b*	0.608	-1.47577455
NM_001081321	*Pds5a*	0.644	-1.03048504
NM_024452	*Luzp1*	0.747	-1.27570871
NM_178661	*Creb3l2*	0.837	-1.08859245
NM_024472	*Cptp*	0.862	-1.27774791
NM_177644	*Rasal2*	1.228	-1.14013994
NM_144515	*Zfp52*	1.257	-1.33247327
NM_028778	*Nuak2*	1.581	-1.20811393
NM_153287	*Csrnp1*	1.748	-1.30599888
NM_026473	*Tubb6*	1.983	-1.15399286
^∗^NM_008120	*Gja4*	3.703	-1.38683764

**FIGURE 4:**
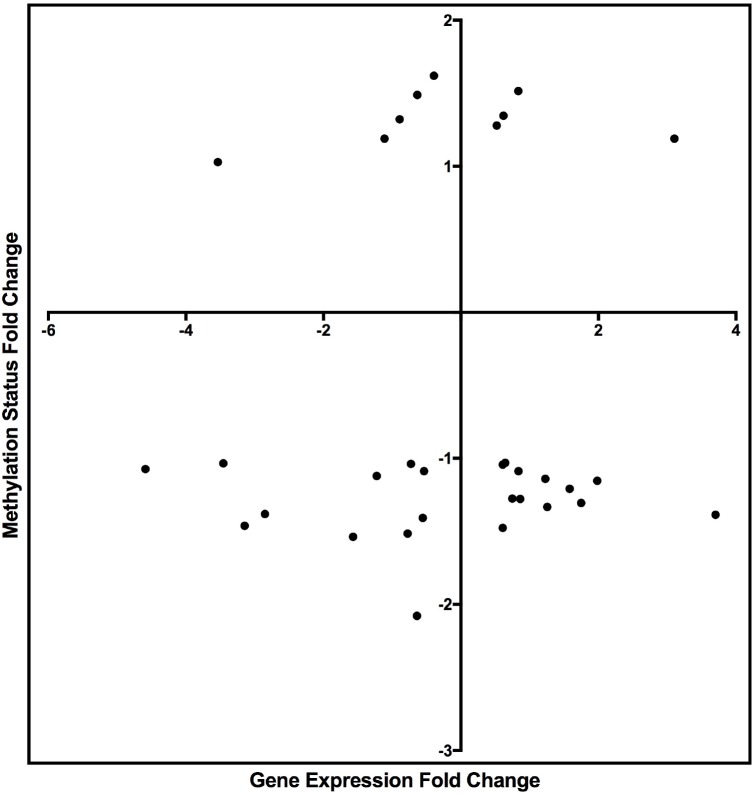
Scatter plot graph of the genes listed in Table 7 by fold change of gene expression and DNA methylation.

*The DE-gene with methylation are listed to show the gene expression is related to methylation, including the gene expression with both positive and negative correlation to DNA methylation. A scatter plot graph shows these genes based on gene expression level and methylation status fold (Figure 4).*

Literature searches were performed for each methylated DE-gene exhibiting a negative correlation to identify the genes that were reportedly regulated by TGF-β. Among the negatively correlated genes, KLF7 expression is regulated by TGF-β, reported by previous study (Wang et al., 2016). The downregulation of KLF7 was likewise negatively associated with DNA hypermethylation, suggesting that DNA methylation plays an important role in KLF7 expression. The results also indicated that the expression of Gja4, or connexin-37, was upregulated by TGF-β, which is consistent with other prior reports (Ota et al., 2002; Jose et al., 2011). Further, the DNA hypomethylation in Gja4 suggested that methylation plays a critical role in the negative regulation of Gja4 expression.

**FIGURE 5:**
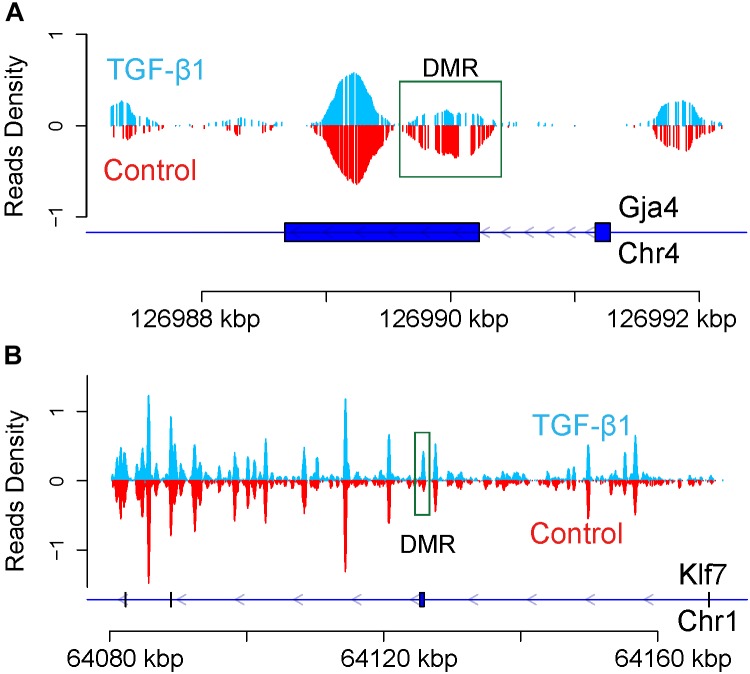
Enrichment of DNA methylation of Gja4 **(A)** and KLF7 **(B)** in regulatory gene region. The blue line represents the gene. The blue box represents CDS. Arrow represents the transcription direction.

To investigate the methylation status further, the DMR of the TGF-β-regulated genes KLF7 and Gja4 were analyzed to determine potential differences in the methylated regions of the genes following TGF-β treatment. Based on a comparison of the read density distributions across the DMR-labeled KLF7 and Gja4 genes between the TGF-β-treated and control samples, we found the hypomethylation in the second exon of Gja4 in the TGF-β-treated sample (Figure 5A), whereas the methylation in the second exon of KLF7 was markedly increased (Figure 5B).

### Pathway Analysis Based on Methylated DE-Genes

To identify the signaling pathways involved in TGF-β1-regulated methylation, the methylated DE-genes were analyzed by IPA (Supplementary Table S6). The results of the top 5 pathway analysis (Table 8) demonstrated that “epithelial adherens junction signaling” was the top pathway, suggesting that cell adherence is regulated by DNA methylation in mesangial cells following TGF-β1 treatment. Other pathways identified included nicotinamide adenine dinucleotide (NADH) repair, ketolysis, ketogenesis, and the mevalonate pathway. Among these methylated DE-genes in these pathways, tubulin beta 6 (TUBB6) expression was negatively correlated to DNA methylation. Tubulin proteins also participate in the TGF-β signaling pathway and are involved in the fibrotic process (Chilosi et al., 2017). Hydroxyacyl-CoA dehydrogenase trifunctional multienzyme complex subunit alpha (HADHA) was also listed among the top pathways (Table 8), which was consistent with a recent study showing that HADHA is involved in the development of type II diabetes via TGF-β regulation (Bohm et al., 2016). Our data indicated that HADHA may participate in TGF-β1-induced diabetes and is associated with DNA methylation that results in positive regulation. Generally, these data indicate that DNA methylation regulates several key genes that are involved in the TGF-β-induced kidney injury process.

**Table 8:** Ingenuity Pathway Analysis pathway analysis of DE-genes with DNA methylation.

Ingenuity canonical pathways	-log(B-H *p*-value)	DE-genes
Epithelial adherens junction signaling	2.82	NOTCH1,AFDN,TUBB6
NADH repair	2.31	GAPDH
Ketogenesis	1.89	HADHA
Ketogenesis	1.79	HADHA
Mevalonate pathway I	1.71	HADHA

### Analysis of H3K27me3 Methylation in Response to TGF-β Treatment

**FIGURE 6:**
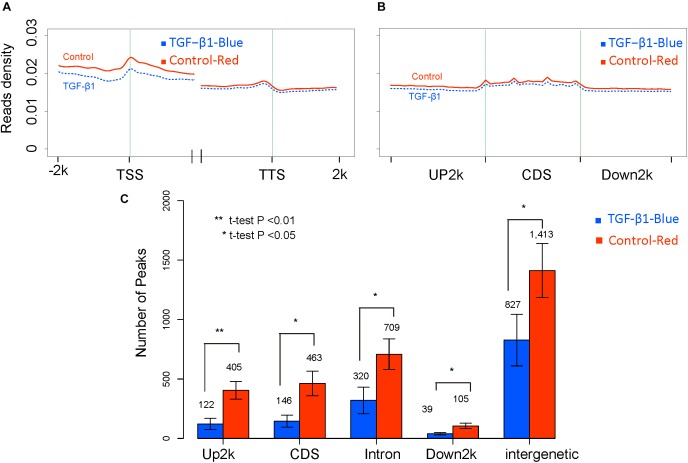
The global H3K27me3 modification level between control and TGF-b samples. **(A)** The reads density in transcription start sites (TTS) and transcription termination sites (TTS). **(B)** The reads density across gene elements, including up2k, down2k and Coding DNA Sequencing (CDS). **(C)** The distribution of peaks through different gene elements.

Trimethylated H3K27 (H3K27me3) has been linked to gene repression (Dong and Weng, 2013) and is regulated by TGF-β1 (Estaras et al., 2013). However, controversial results have revealed an opposing role of H3K27me3 in kidney fibrosis (Siddiqi et al., 2016; Zhou et al., 2016). To clarify the modification of H3K27me3 in response to TGF-β1, we normalized read distributions into read densities using the method applied in the DNA methylation study. The average read density of H3K27me3 levels was then analyzed across the entire genome in regions adjacent to TSS and TTS (Figure 6A), and in the Up2k, Down2k, and CDS gene elements (Figure 6B), which revealed that TGF-β1 treatment might reduce H3K27me3 levels in the genome. We then explored the peak distributions on different gene elements in each biological replicate and found significant differences in the peak numbers (Figure 6C). Our results suggest a significant reduction in H3K27me3 levels following TGF-β treatment, which is consistent with previous reports (Zhou et al., 2011). Next, we focused on the analysis of individual genes exhibiting H3K27me3 modifications to identify the key genes regulated by histone modification.

### Integrative Analysis Between H3K27me3 Modified Genes and DE-Genes

An analysis of differentially histone methylation regions (DHMRs) identified 40 genes with differential H3K27me3 modification, including 7 genes that were hypermethylated and 33 genes that were hypomethylated after TGF-β treatment (Supplementary Table S7). After analyzing the relationship between H3K27me3 levels and gene expression, 15 DE-genes with DHMRs were identified (Table 9). Based on a negative correlation analysis, we identified 6 DE-genes with H3K27me3 modification in the TGF-β treatment group, one of which was downregulated and hypermethylated and others that were upregulated and hypomethylated.

**Table 9:** Integrative analysis between H3K27me3 modification and DE-genes.

Gene ID	Symbol	Gene expression fold change	H3K27me3 fold change
**Positively correlated**		
NM_010074	Dpp4	-16.003	-1.34722077
NM_172463	*Sned1*	-6.257	-1.16715932
NM_008259	*Foxa1*	-5.241	-1.82107269
NM_175473	*Fras1*	-5.204	-1.19562114
NM_021331	G6pc2	-4.957	-1.10028594
NM_010501	*Ifit3*	-4.716	-1.78884336
NM_028894	*Lonrf3*	-4.574	-1.54787003
NM_001025570	*Prrx1*	-2.683	-1.28457282
NM_001163098	*Tchh*	-1.47	-1.43213155
**Negatively correlated**		
NM_031176	*Tnxb*	-3.899	1.30043346
NM_007467	*Aplp1*	1.128	-1.05803508
NM_010118	*Egr2*	1.409	-1.99831957
NM_025980	*Nrarp*	5.588	-1.96283384
NM_001008793	Whrn	12.943	-2.75133723
NM_021279	*Wnt1*	13.997	-1.10023151

**FIGURE 7:**
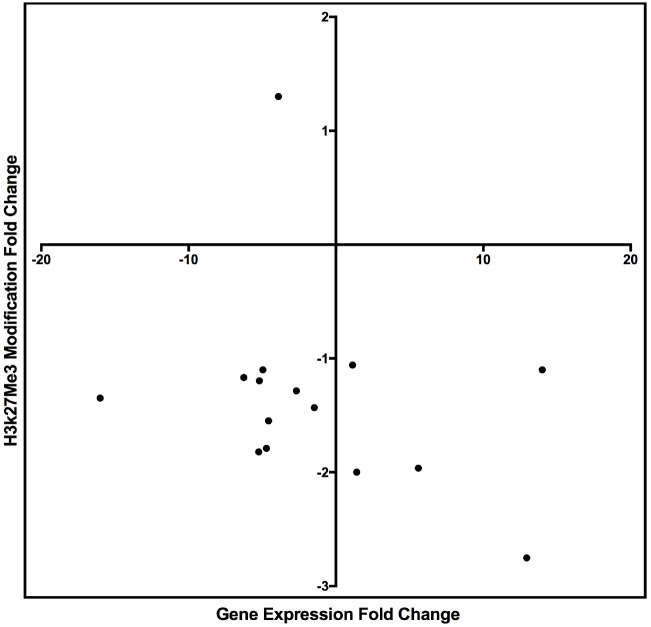
Scatter plot graph of the genes listed in Table 9 by fold change of gene expression and H3K27me3 modification.

*The DE-gene with H3K27me3 modification are listed to show the gene expression is related to H3K27me3, including the gene expression with positive and negative correlation to H3K27me3. A scatter plot graph shows these genes based on gene expression level and methylation status fold (Figure 7).*

Tenascin XB (TNXB), which could be linked to the activation of TGF-β1 (Alcaraz et al., 2014), was the only DE-gene that was downregulated and hypermethylated (Table 9). The remainder of the DE-genes that were upregulated and hypomethylated following TGF-β1 treatment included amyloid-like protein 1 (APLP1), early growth response 2 (EGR2), NOTCH regulated ankyrin repeat protein (NRARP), Whrn, and WNT1 (Table 9). WNT1 is a known fibrotic regulator in TGF-β1-induced pathogenesis (He et al., 2009). Together, our data suggested that TGF-β1-induced Wnt1 expression is regulated by hypomethylated H3K27me3, which acts as a novel mechanism for the Wnt signaling pathway during TGF-β1-regulated mesangial cell damage. These findings are consistent with a previous publication showing the enrichment of histone H3K27 methyltransferase Ezh2 on Wnt genes, which regulated Wnt/β-catenin signaling (Wang et al., 2010). Enhancer of zeste homolog 2 (Ezh2) is the histone methyltransferase of the polycomb repressive complex 2 catalyzing histone H3 lysine 27 tri-methylation (H3K27me3). Deletion of Ezh2 eliminates H3K27me3 and derepresses *Wnt* expression.

**FIGURE 8:**
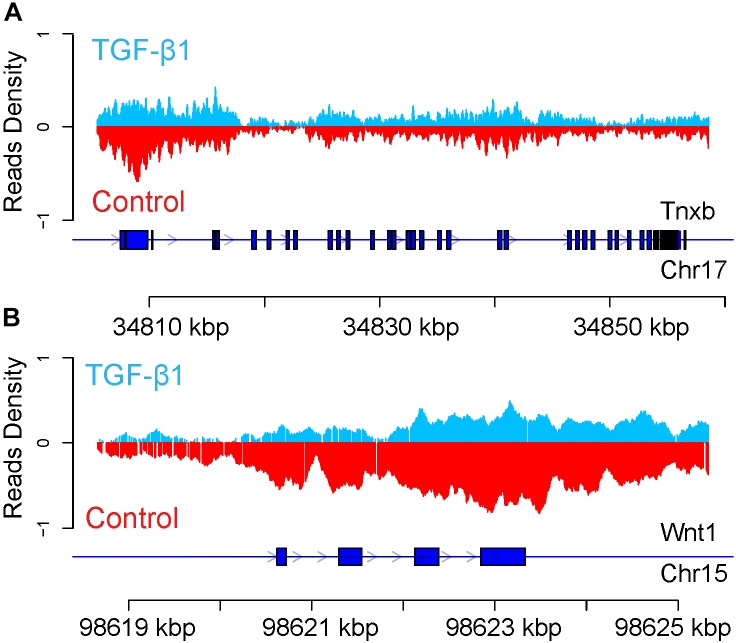
Enrichment of H3K27me3 modification of Tnxb **(A)** and Wnt1 **(B)** in regulatory gene region. The blue line represents the gene. The blue box represents CDS. Arrow represents the transcription direction.

The H3K27me3 peak distribution in the regulatory region of TNXB and WNT1 is shown in Figure 8. We found hypermethylated H3K27me3 peaks in TNXB after TGF-β1 treatment compared to control samples (Figure 8A) and hypomethylated H3K27me3 on WNT1 in TGF-β1-treated samples (Figure 8B). Due to the limited number of genes (12 genes), however, it was not feasible to proceed to a pathway analysis.

More interestingly, the comparison of the list of differentially methylated genes between DNA methylated (Table 7) and H3K27me3 modified (Table 9) revealed no overlapping genes, suggesting that TGF-β1 induced a singular epigenetic change between DNA methylation and H3K27me3 modification.

## Discussion

This study combined NGS-based discovery and characterization, which enabled integrative transcriptome, small RNA, and epigenomic analyses across all genetic variations according to the following framework: (i) complementary functional analyses, which facilitated the identification and validation of novel regulators and genes involved in signaling pathways activated during kidney injury; and (ii) integrative analyses between miRNAs, RNAs, DNA methylation, and H3K27me3 modification to explore the mechanistic regulation of gene expression.

The cytokine TGF-β1 plays critical roles in kidney development and inflammation. However, relying on traditional microarray screening during different disease states to identify potential regulators has resulted in the identification of only a limited number of target genes. Moreover, epigenetic modifications and corresponding downstream signaling proteins cannot be identified by investigating the functions of single genes. To overcome these limitations, we constructed the first global cellular characterization at various molecular levels, to identify genomic sequences and signatures in kidney cells that are unique to TGF-β1. Thus, this work is a first empirical characterization of the mechanisms through which 3 days of TGF-β exposure alters gene expression. Future work, both experimental and theoretical, is required to uncover the underlying genetic networks.

Mesangial cells constitute the central stalk of the kidney glomerulus. Accordingly, mesangial pathology plays a central role in several kidney diseases that are recognized as major health burdens worldwide. Our work based on TGF-β1 as a master regulator of kidney mesangial cell injury will help identify additional candidate regulators, which facilitate the development of improved strategies for the treatment of tissue fibrosis in other organs, in turn, to enhance disease prevention and drug discovery efforts on a global scale.

Based on the expression patterns detected, we generated a cellular characterization that linked transcriptional factors, small RNA, and epigenetic factors. First, it was critical to identify genes that were altered in response to TGF-β1 treatment, recognizing that the simple FC in gene expression yields under representative data that does not accurately reflect the importance of some genes. Conversely, determining the highly expressed endogenous genes in control cells that exhibited reduced expression following TGF-β1 treatment was more informative. Similarly, the genes with low expression levels in control cells that were highly expressed in TGF-β1-treated samples were also potential candidates in the regulation of TGF-β1-induced damage.

The principal advantage of the miRNA-RNA target relationship was a reduction in the large number of predicted targets following the use of miRNA target analysis tools. Although we were unable to identify the direct targets affected by inhibition of translation inhibition, we were able to identify the gene targets based on mRNA expression levels. It is known that miRNAs repress the 3′UTR of target genes at the translational level. Theoretically, miRNA upregulation inhibits the expression of target genes; thus, the downregulation of miRNA might conversely induce the expression of target genes. To narrow the target gene selection, we compared the predicted target genes to the gene expression patterns to identify the upregulated and downregulated target genes, which enabled us to construct a gene expression map that demonstrated the expression levels of genes in response to TGF-β1, providing a useful tool for more informative gene selection. Therefore, this project focused on the repressive transcription function of miRNA. More recent evidence reports a more regulatory function of miRNA involved in guided gene regulation (Catalanotto et al., 2016).

The quintile classification of endogenous DE-gene expression demonstrated an alternative analysis method to FC. Using quintile classification, we noted that a series of DE-genes that exhibited a significant change in endogenous distribution were involved in TGF-β regulation. Conversely, cadherin 10 (Cdh10), which acts as a biomarker of TGF-β-induced epithelial-mesenchymal transition (Johansson et al., 2015), exhibited low expression levels (Rank 1) in control samples, but was highly expressed (Rank 4) following TGF-β treatment. Another example is Trib3, a known promoter of TGF-β induced fibrosis (Tomcik et al., 2016), which likewise exhibited low expression levels (Rank 1) in control samples, but was highly expressed (Rank 4) following TGF-β treatment.

DNA methylation is known to be regulated by TGF-β1 in different cell lines. However, the number of altered genes that are regulated by DNA methylation remains unclear. Generally, DNA methylation induces gene silencing. To determine the critical function of DNA methylation in gene expression, we compared the genes exhibiting DNA methylation to negatively correlated DE-genes regarding expression level. Interestingly, we identified several DE-genes that play important roles in the development of TGF-β1-regulated fibrosis via DNA methylation. However, many DE-genes exhibited a positive correlation to DNA methylation, suggesting that other transcriptional factors influence final gene expression levels. Our data showed the expression level of KLF7 and Gja4 are linked to DNA methylation, suggesting that this is the main mechanism through which TGF-β1 regulates these two genes. Further experiments will be designed to assay DNA methylation level in the DMR, including the promoter activity in response to TGF-β1.

Histone modification is another important regulatory factor that contributes to kidney injury. EZH2 induces the expression of H3K27me3, which, in turn, regulates the development of kidney fibrosis. However, recent studies have shown an opposing function of H3K27me3. Our results demonstrated that the overall H3K27me3 expression level was altered following TGF-β1 treatment compared to control samples. The gene expression map was also compared to the genes exhibiting H3K27me3 modifications. Interestingly, the recognized fibrotic factor wnt1 was induced by TGF-β1 corresponding to H3K27me3 modification, which suggested that H3K27me3 may play an important role in kidney fibrosis. Further, the controversial function of H3K27me3 in kidney fibrosis might be due to the use of different kidney injury models or differing responses to specific cells. Our results in TGF-β1-induced mesangial cells, however, showed an overall downregulation of H3K27me3, which was consistent with a previous *in vivo* study of diabetic nephropathy that reported a protective role of H3K27me3 in chronic kidney injury (Siddiqi et al., 2016). Interestingly, we did not detect any DE-genes that were DNA methylated and exhibited H3K27me3 modifications, suggesting the singular occurrence of methylation in single gene.

We describe the first comprehensive study to confirm the existence of DNA methylation and H3K27me3 in response to TGF-β1 treatment. Our data also provides evidence of the association between epigenetic changes and the expression of genes that are closely related to kidney injury. In this context, future studies should examine processes in which epigenetics is involved in the suppression of the spurious transcription initiation of kidney injury-related genes, which could facilitate the discovery of new mechanisms underlying kidney fibrosis.

Overall, our discoveries enhance the current understanding of the alternative mechanisms that contribute to the expression of TGF-β1-regulated genes involved in the pathogenesis of kidney injury. This study likewise offers insight into the molecular underpinnings of TGF-β1 stimulation in kidney cells and provides a robust platform for further target exploration.

## Author Contributions

BW designed the project, analyzed the data, and wrote the manuscript. SR supervised the project and finalized the manuscript. GJ and HN helped analysis of the data. The remaining authors provided technical support and instruction to this project.

## Conflict of Interest Statement

GJ and JW are employed by Shenzhen E-GENE Tech Co., Ltd. The remaining authors declare that the research was conducted in the absence of any commercial or financial relationships that could be construed as a potential conflict of interest.

## Supplementary Material

TABLE S1 The full list of differentially expressed gene sets between control and TGF-β samples.

TABLE S2 The canonical pathway by IPA analysis of differentially expressed genes. -log(B-H *p*-value) is -1 ^∗^ log(*p*-value) after Benjamini-Hochberg multiple hypothesis correction. The *z*-scores are “activation *z*-scores” as calculated by IPA canonical pathways analysis, as described in Kramer et al. (2014). Empty values are pathways where IPA was unable to calculate a *z*-score. The ratio is the proportion of selected genes hitting a given pathway, out of the total genes in that pathway that were considered in the experiment.

TABLE S3 The ranking list of differentially gene expression between control and TGF-β samples.

TABLE S4 The list of DE-miRNA and their target DE-genes.

TABLE S5 The genes with DNA methylation between control and TGF-β samples.

TABLE S6 The canonical pathway by IPA analysis of differently expressed genes with DNA methylation.

TABLE S7 The genes with H3K27me3 modification between control and TGF-β samples.
